# A Deep Learning Model for 3D Ground Reaction Force Estimation Using Shoes with Three Uniaxial Load Cells

**DOI:** 10.3390/s23073428

**Published:** 2023-03-24

**Authors:** Junggil Kim, Hyeon Kang, Seulgi Lee, Jinseung Choi, Gyerae Tack

**Affiliations:** 1 Department of Biomedical Engineering, Konkuk University, Chungju 27478, Republic of Korea; 2 BK21 Plus Research Institute of Biomedical Engineering, Konkuk University, Chungju 27478, Republic of Korea

**Keywords:** three-axis ground reaction force estimation, seq2seq LSTM, load cell, gait

## Abstract

Ground reaction force (GRF) is essential for estimating muscle strength and joint torque in inverse dynamic analysis. Typically, it is measured using a force plate. However, force plates have spatial limitations, and studies of gaits involve numerous steps and thus require a large number of force plates, which is disadvantageous. To overcome these challenges, we developed a deep learning model for estimating three-axis GRF utilizing shoes with three uniaxial load cells. GRF data were collected from 81 people as they walked on two force plates while wearing shoes with three load cells. The three-axis GRF was calculated using a seq2seq approach based on long short-term memory (LSTM). To conduct the learning, validation, and testing, random selection was performed based on the subjects. The 60 selected participants were divided as follows: 37 were in the training set, 12 were in the validation set, and 11 were in the test set. The estimated GRF matched the force plate-measured GRF with correlation coefficients of 0.97, 0.96, and 0.90 and root mean square errors of 65.12 N, 15.50 N, and 9.83 N for the vertical, anterior–posterior, and medial–lateral directions, respectively, and there was a mid-stance timing error of 5.61% in the test dataset. A Bland–Altman analysis showed good agreement for the maximum vertical GRF. The proposed shoe with three uniaxial load cells and seq2seq LSTM can be utilized for estimating the 3D GRF in an outdoor environment with level ground and/or for gait research in which the subject takes several steps at their preferred walking speed, and hence can supply crucial data for a basic inverse dynamic analysis.

## 1. Introduction

Walking is a fundamental human action; it is accomplished by a force that alters the foot’s position on the ground [1]. Understanding the kinetic and kinematic interactions between the human body and the ground requires GRF (ground reaction force) [2]. GRF is the force exerted by the ground on a body in contact with it. A force plate is a measurement platform, each corner of which has a piezoelectric force sensor. Due to the precision of their measuring results, force plates have long been considered the gold standard. However, if GRF is measured using a force plate, one force plate is required for each step. As a result, as the number of steps to be observed increases, additional force plates are necessary, and this is one of the limitations of force plates. Pressure mats, such as GAITRite (CIR Systems Inc., Clifton, NJ, USA), Walkway and Stance Pad (XSENSOR Inc., Calgary, AB, Canada), and Strideway System (Tekscan Inc., Norwood, MA, USA), can be utilized to measure the vertical pressure value for each step across lengths exceeding five meters [3,4,5]. Using 3000 pressure sensor arrays along a length of about seven meters, GAITRite can record foot pressure during the stance phase of a gait [6]. Using collected data to calculate gait characteristics, such as step length, step velocity, single or double stance duration, and cadence, it can provide a quantitative gait evaluation [7]. With the use of these pressure mats, only a vertical GRF evaluation is possible. The calculation of the inverse dynamics of actual joints requires a three-dimensional GRF. However, a 3D GRF cannot be constructed using data from a single vertical axis.

Recent research [8,9,10] indicates that investigations outside the laboratory are required to fully comprehend walking. Like force plates, pressure mats can only be utilized in a laboratory setting, despite their portability. Inserting a pressure insole into the shoe is the most popular method for measuring GRF in an outdoor setting. A force-sensitive resistor (FSR) is typically employed as the insole sensor [5,11]. The FSR is placed into the shoe in an array to detect changes in foot pressure and calculate the pressure value along the vertical axis. In recent years, numerous studies [8,12,13] have investigated the use of FSR-based guidance when walking outside the laboratory.

There have been considerable efforts to estimate GRF using sensor data. Using 99-FSR-array measurement data, Honert et al. computed the GRFs in the anterior–posterior (AP) and vertical directions during walking [8]. This work encouraged researchers who estimate GRFs outside the laboratory, as it accounted for the irregular ground environment while walking outside by learning individual models dependent on a slope. Choi et al. estimated vertical GRF using six FSRs [14] and demonstrated that it is possible to estimate vertical GRF with high precision using only six FSR sensors rather than an array of FSR sensors. Oubre et al. [15] also estimated the three-axis GRF by using six FSRs. In [14,15], six FSRs were used to measure the pressure values of various foot regions, and the participant manually positioned each sensor in a predetermined location.

Many applications in the external environment and long-term walking studies have been undertaken using FSRs, but the following limitations have been identified: (i) The flexibility of FSR arrays causes the sensor response to be distorted depending on the deformation of the inner contact surfaces of the shoe and foot under pressure [16]. (ii) The load imposed as a result of the heat inside the shoes causes drift [16]. (iii) Due to the nonlinear nature of the pressure–resistance response, user- and sensor-specific calibration are necessary [17]. Additionally, the values obtained by FSRs and vertical GRFs are somewhat different. Vertical pressures via FSRs can be determined by adding all of the pressure values measured by each FSR array cell. Despite the high correlation with vertical GRF, there is a discrepancy in the absolute values since the calculated vertical pressure values are vertical pressures measured by the sensor itself. When the angle between the ground and the foot deviates from 90° as the foot moves from the heel-strike to the toe-off, the pressure detected by the sensor and the vertical GRF will diverge more [18].

By replacing an FSR with a load cell, several issues caused by FSR flexibility can be resolved. As the load cell is made of a rigid material, such as aluminum, there is no noise caused by the sensor’s distortion while walking. In addition, unlike FSRs, load cells can measure three-axial forces, enabling the direct calculation of the 3D GRF from the measurement. Tao et al. [19] calculated the 3D GRF and CoP (Center of Pressure) using two three-axial load cells while walking. In addition, this study provided guidelines for quantitatively assessing GRF during various rehabilitation walking conditions. Zullo et al. also calculated a 3D GRF using two three-axial load cells, although this was not a study of walking [20]. It is possible to obtain GRF directly with high accuracy using three-axial load cells; however, the economic efficiency of three-axial load cells is somewhat limited because they are more expensive than FSRs. Uniaxial load cells can be used to match the economic efficiency and usability of FSRs as well as the durability of load cells. It was difficult to find previous studies that calculated or estimated 3D GRF using uniaxial load cells.

Recent advancements in artificial intelligence have increased the precision of signal prediction models, which forecast output signals by utilizing complex input signals. This signal prediction model [21,22] can replace the general approach in the field of biomechanics for calculating muscle forces and/or joint moments using measured GRF data and equations of motion, such as Newton–Euler or Euler–Lagrange. Seq2seq is a method for transforming one input signal into another that is mostly utilized for natural language processing tasks such as text summarization and translation [23,24]. Seq2seq is carried out by means of a recurrent neural network (RNN) [25,26] specializing in time series modeling. This model, introduced in the 1980s, is a neural network that generates output data by mixing input data with a hidden state that relies on prior time steps and has the benefit of learning by taking into account temporal correlations between events in time series data [27]. An RNN observes a problem when a weight is not updated successfully in repeated backpropagation when the number of hidden layers increases, when computing output data, or when the correlation is predominantly applied to the result of a step distance from the current step [27,28]. Long short-term memory (LSTM), an alternative neural network for RNNs, adds a four-layer cell state to the RNN’s hidden state in order to effectively tackle gradient vanishing [28,29].

In order to alleviate the disadvantages of FSRs and boost their future usefulness, a uniaxial load cell was utilized. For this study, we built a deep learning model, seq2seq LSTM, to estimate three-axis GRF utilizing shoes with three load cells. The correlation coefficient and RMSE between the calculated and actual GRFs and the Bland–Altman analysis for the maximum values of the GRFs [30] were used to validate the final LSTM model.

## 2. Materials and Methods

### 2.1. Shoe with Three Uniaxial Load Cells

Three optical uniaxial EzForce-1D (i2A Systems Inc., Daejeon, Republic of Korea) load cells were used to measure GRFs during walking [31]. The optical load cells do not need a voltage amplifier and can be miniaturized by measuring the displacement of the elastic body. The characteristics of the load cells used are shown in Table 1.

Due to the load cell’s aluminum construction, it is durable, and the distortion of the response signal created by the FSR may be minimized [16,17]. To prevent sensor alterations from occurring within the insoles of the shoes, the system was built with sensor-integrated footwear. Three load cells were installed as follows: one was placed on the 1st and one on the 5th metatarsal bone, and one was placed on the mid-heel bone. This configuration was developed to identify the temporal event of a change in the center of pressure while walking as efficiently as possible using only three sensors [32,33]. In addition, shoes with load cells were constructed so that the size could be adjusted based on the subject’s foot size, thus reducing experimental bias.

**Figure 1:**
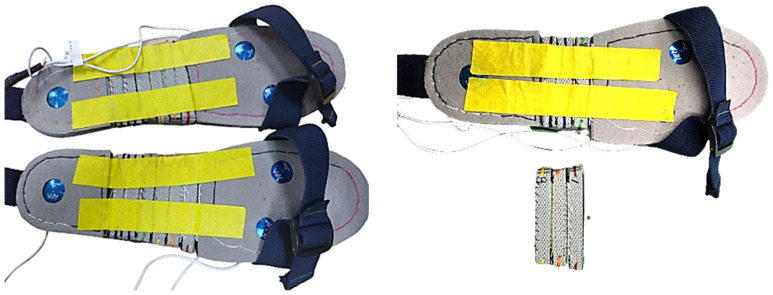
Developed shoes with three uniaxial load cells. By removing the middle blocks, the overall shoe size can be adjusted to precisely position the sensor on the subject’s heel and their 1st and 5th metatarsal. One block is approximately 10 mm wide.

As each load cell had a height of 6.9 mm, four nonwoven fabrics with a thickness of 2 mm were layered, followed by the load cells. To allow the sensor to be positioned on the subject’s heel and metatarsal bones based on the subject’s foot size, Velcro was used to attach blocks with a 1 cm spacing. Figure 1 shows the developed shoe with three load cells. MyRIO-1900 (National Instruments Corporation) was used to acquire load cell data [34]. MyRIO is equipped with a dual-core ARM Cortex-A9 processor, an artix-7 field programmable gate array (FPGA), and hardware support for algorithms based on the Xilinx Zynq-7010 SoC, offering the performance required to construct complicated real-world systems [34]. Analog load cell data were wired to MyRIO and were delivered to the PC via WiFi at a 100 Hz sampling frequency in MyRIO. A Vi program based on LabView 2014 (National Instruments Corp., Austin, TX, USA) was used to collect and store the data, and the converted force value was saved in a .csv file to reflect the load cell’s voltage–force coefficient.

### 2.2. Experiment

The study included 81 healthy young people (40 men (age: 23.44 ± 2.18) and 41 women (age: 23.33 ± 2.21)). The protocol of this study was approved by the Ethics Committee of our university (see Institutional Review Board Statement). The purpose and methodology of the study were fully explained to all the participants who were enrolled in the study after they provided their informed consent. Each subject walked 7 m at their preferred speed while wearing shoes equipped with three load cells that were tailored according to their unique foot size. While demonstrating their gait, subjects stepped on two buried force plates (Kistler Instrumente AG, Winterthur, Switzerland), and the GRF was measured at a sampling frequency of 120 Hz using Cortex 4.0. (Motion Analysis Corp., Rohnert Park, CA, USA). The experiment was carried out twice, with the subjects stepping on two of the four force plates positioned along the 2 m course.

### 2.3. Data Processing

**Figure 2:**
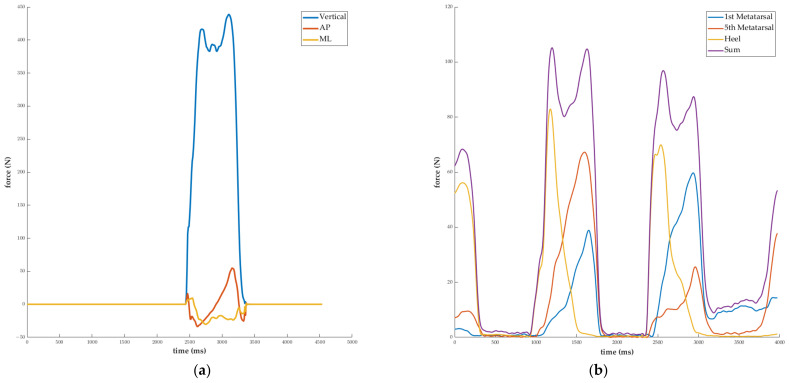
Typical signal from force plate and load cells. (**a**) Raw GRF measured from force plate. (**b**) Signal measured from three load cells and sum of three load cells during walking.

Only 109 of the 162 sets of data collected from the 81 participants with both feet in full contact with two force plates were selected for the final dataset. Twenty-one of the individuals failed to accurately step on the force plate in both gait studies. Because the measurement sampling frequencies of the load cell data and GRF data were 100 and 120 Hz, respectively, all of the data were resampled at 200 Hz. In the case of the load cell data, the signal was continuously monitored during all 7 m sections of the walking experiment, whereas the force plate data were only recorded for a single step for each foot. Therefore, only the load cell data collected at the time of force plate contact during walking were chosen. A fourth-order Butterworth lowpass filter with a cutoff frequency of 15 Hz was applied to the signal to reduce noise. At the heel-strike point, the filter signal was synced. The heel-strike was defined as the point at which the vertical force occurred for the force plate data, and it was defined as the point at which the heel force occurred for the load cell data. During the swing phase of walking, the force plate data were always taken at 0 N, making it simple to determine the heel-strike. Since the load cell sensor always makes contact with the foot, even during the swing phase, very small values were recorded for the load cell data. When resampling and filtering were conducted, these noises were converted to close to zero, and the identical heel-strike as the force plate could be determined. All of the data analysis was accomplished using MATLAB 2022a (Mathworks, Nantick, MA, USA). Figure 2a shows typical force plate signals, while Figure 2b shows typical load cell signals.

### 2.4. LSTM Seq2seq Layer

**Figure 3:**
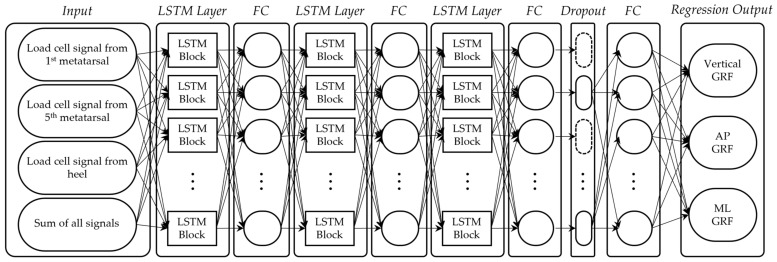
Seq2seq LSTM layer to estimate 3D GRF.

Seq2seq LSTM based on the MATLAB deep learning toolbox was used to forecast the GRF when walking. Four signals from three distinct foot regions and their sum (Figure 2b), as recorded by three load cells, were set as the input. The vertical, AP, and ML GRFs measured by the force plate were set as the output (Figure 2a). The neural network consisted of three LSTM layers (number of nodes: 200, 50, 200), with each layer connected to a fully connected (FC) layer, followed by a regression layer that generated three-axis GRFs. The epochs were 1000, the optimizer was Adam, the learning rate was 0.01, and the minibatch size was 32 during training. We have included the parameter details in the Supplementary Material. Additionally, we have provided information on the computer specifications used in our study and the cost of training the model. These details can be found in the Supplementary Material as well. Figure 3 shows the seq2seq LSTM layer.

### 2.5. Validation

To conduct the learning, validation, and testing, random selection was performed based on the subjects. The 60 selected participants were divided as follows: 37 in the training set, 12 in the validation set, and 11 in the test set. Since data from the same subjects were not divided into training, validation, and test datasets, this approach ensured that the test set was independent from the other two sets; thus, this model does not require new training for new subjects. The training (32), validation (8), and test (9) datasets were chosen for the real dataset following the success of the two experiments. The root mean square error (RMSE) and correlation coefficient between the measured and predicted GRFs for the test set were calculated to evaluate the performance of the trained model [8,14,15,35]. The timing error was determined when the AP GRF value changed from negative to positive, i.e., from the braking phase to the propelling phase [35]. This illustrated the error probability of the gait timing variable when the gait analysis was performed using the estimated ground reaction force. In addition, to confirm the agreement more clearly between the estimated GRF and the measured GRF, a Bland–Altman analysis was performed on the maximum GRF values [30]. A Bland–Altman analysis refers to the use of a Bland–Altman plot, a scatterplot that clearly demonstrates the degree of agreement between two measurements. The X-axis of the graph represents the average of the two measurements, while the Y-axis indicates the difference. The Bland–Altman Plot displays four results, each of which is defined as follows: (i) bias: the mean of the difference between two measurements; (ii) 95% confidence interval (CI) of bias: an indication of the magnitude of the systematic difference; (iii) limits of agreement (LoA): 95% CI of the difference between the two measurements, which serves as the basis for determining the consistency between the two measurements; (iv) 95% CI of LoA: an indication of the error or precision of the upper and lower LoA limits.

## 3. Results

**Figure 4:**
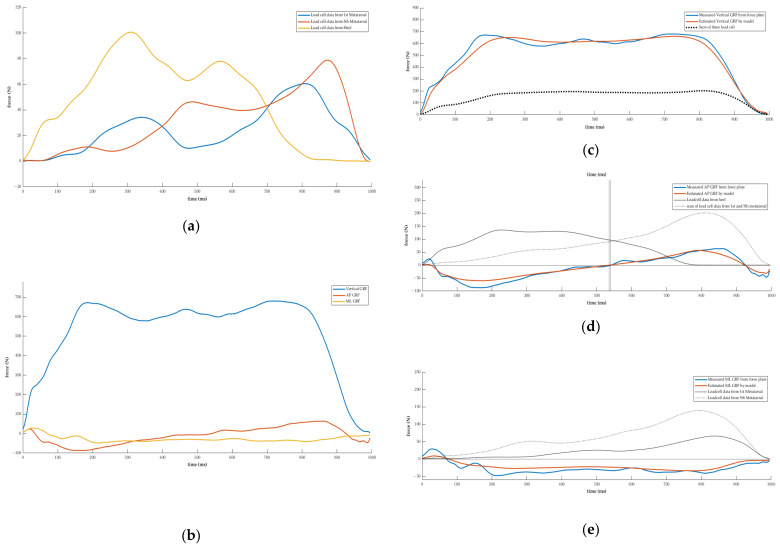
Example signals. (**a**) Measured signals from load cells, i.e., input signals for model. (**b**) Measured GRF from force plate, i.e., output signals for model. (**c**) Vertical GRF measured from force plate and estimated by model. (**d**) AP GRF measured from force plate and estimated by model. (**e**) ML GRF measured from force plate and estimated by model.

Figure 4a,b show a typical example of the measured load cell signals utilized as inputs and outputs of the LSTM, while Figure 4c–e show the three-axis GRF signals predicted by the LSTM, illustrating the similarities between the predicted and measured data. The time when the AP GRF transitions from negative to positive is shown by vertical lines in Figure 4d. The difference between the predicted and actual values is 0.005 s in this example.

The correlation coefficients between the measured GRF and the predicted GRF, the RMSEs, and the mid-stance timing error are shown in Table 2. The vertical, AP, and ML GRF correlation coefficients were 0.97, 0.96, and 0.90, respectively, while the RMSEs were 65.12 N, 15.50 N, and 9.83 N, respectively, and mid-stance timing error was 0.06 s. In nine out of nineteen cases, the projected AP GRF was faster than the measured AP GRF, in ten cases it was slower, and in one case it was the same.

**Figure 5:**
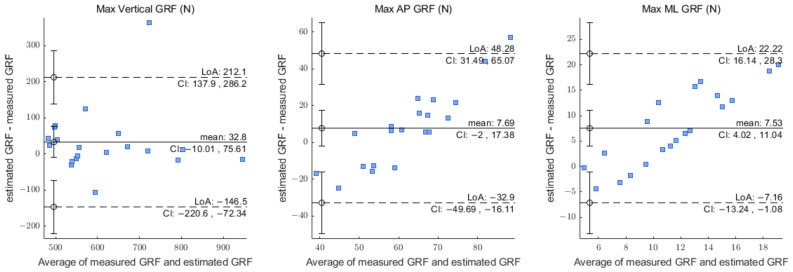
Bland–Altman plots of the estimated GRF vs. measured GRF (max vertical, AP, and ML). The straight line and the dotted line represent the bias and SD limits.

Figure 5 is a Bland–Altman plot for max vertical, AP, and ML GRF. It shows that the majority of max 3D GRFs were distributed within the LoA. This indicates that the estimated GRF closely matches the actual GRF. Only one datum from the Max Vertical GRF deviated from the LoA 95% CI. A detailed summary of the results regarding the mean difference and limits of agreement can be seen in Table 3.

## 4. Discussion

Ground reaction force is a crucial variable for inverse dynamic analysis, a technique utilized to determine the internal forces and moments acting on the body based on the motion and external forces applied to the body. Three-dimensional GRF is vital for the calculation of the internal forces and moments, which in turn are essential for understanding the biomechanics of human movement. Consequently, analyzing GRFs provides researchers with valuable insights into the mechanisms underlying human movements. In this study, we aimed to estimate the trajectory of 3D GRFs using shoes equipped with three uniaxial load cells and a sequence-to-sequence LSTM model.

In this study, three-axis GRFs were predicted using seq2seq LSTM with 109 datasets obtained from 81 healthy adults as they walked with shoes outfitted with three load cells. For the RMSEs, verification with test sets showed vertical results of 65.12 N, AP:15.50 N, and ML:9.83 N, which are similar to the vertical results of 57.2 N, AP:20.8 N, and ML:10.1 N obtained by Oubre et al. [15]. Fong et al. [36] reported vertical results of 56.20 N, AP:41.60 N, and ML:7.55 N, and F Wei [37] reported vertical results of 23.95 N, AP:20.12 N, and ML:10.05 N when the GRF was predicted using a Pedar with 99 FSRs and linear regression. In the above two studies, feature selection was performed during linear regression, and in the study by Fong et al. [36], two sensors in the metatarsal region and two sensors in the heel region were ultimately selected, a configuration similar to the load cell insertion position used in our study, and the RMSE was nearly equivalent. In our study, it was shown that the correlation coefficient for the vertical and AP GRFs was at least 0.96. This is greater than the 0.94 obtained by Choi et al. [14], who predicted the vertical GRF with the use of six FSRs, and the 0.94 obtained by Oubre et al. [15]. It is also greater than the results obtained by Honert et al. [8] (vertical: 0.83 and AP: 0.91), achieved by measuring the pressure of the entire foot using a Pedar.

**Figure 6:**
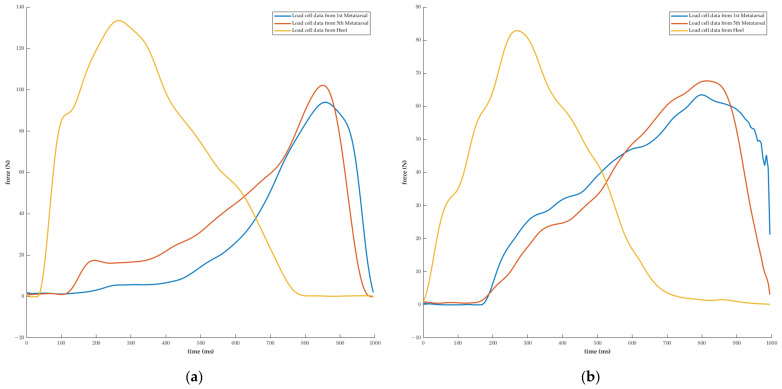
Difference in accuracy between the 1st and 5th metatarsal according to the subject’s gait characteristics. (**a**) The start of pressure on the 5th metatarsal occurred before the start of pressure on the 1st metatarsal. (**b**) The start of pressure on the 5th metatarsal and the start of pressure on the 1st metatarsal occurred at the same time.

As the correlation coefficient between the vertical GRF recorded from a force plate and the simple sum of each load cell measurement was 0.84, it was anticipated that the accuracy of the predictions would be relatively high. We expected that the AP GRF would be predicted based on the difference between the values measured in 1st metatarsal, the 5th metatarsal, and the heel, as well as the occurrence time, whereas we expected that the ML GRF would be predicted based on the difference between the values measured in the 1st metatarsal and 5th metatarsal, as well as the occurrence time. All three-axis predictions confirmed a high degree of accuracy, but the correlation coefficient in the ML direction was relatively low at 0.90. In general, the change in the center of pressure applied to the foot during walking begins at the heel, proceeds up the lateral region to the metatarsal region, and ends at the big toe [38,39]. Therefore, the 5th metatarsal pressure generation occurs prior to the 1st metatarsal pressure generation during normal walking [40]. However, in certain subjects who participated in this study, the 1st metatarsal and 5th metatarsal pressure occurred simultaneously (Figure 6). The accuracy of groups with similar timing and with different timing on a test dataset was evaluated. Table 4 showed that the group with similar timing had slightly lower correlation coefficients and higher RMSE values compared to the group with different timing.

Although some subjects demonstrated walking characteristics similar to those of an in-toeing gait, it would have been challenging to confirm this with the time difference between the inner and outer metatarsals alone. In the case of a severe in-toeing gait, in which the foot rotates inward by more than 7°, the 1st metatarsal typically experiences pressure first [40]. In this investigation, the participants were healthy individuals in their twenties; therefore, it is likely that pressure measurement in the metatarsal alone could not reliably detect internal rotation. In contrast, the accuracy of the ML GRFs in this investigation was only marginally inferior to that of the vertical and AP GRFs, and it was comparable to or greater than the values provided in earlier studies.

In the case of AP GRF, the difference between the measured and estimated mid-stance time values was 0.06 s. The research on GRF estimation is very broad; however, we believe that there is no biomechanical study that confirms the timing error in mid-stance as described above. However, for inverse dynamics analysis, the negative and positive GRF values are expected to have a considerable effect on the calculation of joint torque [2], making quantitative evaluation crucial. In a typical gait cycle, the stance phase accounts for 60% [2,41], and the average duration of the stance phase was 0.59 s in our study. Therefore, the min-stance timing inaccuracy for the full gait cycle is 5.61% and 9.32% based just on the stance time. Given the error range of commercial force plates, such as Kistler and AMTI (2% and 1%, respectively), and the error range of the Wii balance board and HUMAC balance system (6% or more [42]), the error range of less than 6% provided in this work is relatively good.

The consistency of the model’s results was evaluated by comparing the maximum values (peak force) of the 3D GRF. In general, the Bland–Altman analysis indicated that the estimated variable and the measured variable for the max vertical GRF were in good agreement. These results guarantee a high level of precision for future data. In one instance, the Max Vertical GRF differed significantly, and there was an instance in which the force on the fifth metatarsal bone was not completely measured. Due to this, it appears that the predicted value of the vertical GRF has been reduced by that value, resulting in a disparity. For the max AP and ML GRFs, there was a trend in the Bland–Altman plot of increasing differences, which induces a relatively broader width of LoA than that of the max vertical GRF.

Using shoes equipped with three load cells and seq2seq LSTM, the three-axis GRF was estimated with high accuracy in this work. The vertical and AP GRF correlations were at least 0.95, while the ML GRF correlations were 0.90, indicating good performance. In addition to the comparison of the measured and estimated GRF values, the mid-stance timing error was also examined.

There have been studies estimating GRFs with significantly higher precision using two three-axial load cells [19,20], but to the best of our knowledge, few studies are based on fewer than three uniaxial load cells. One of the objectives of our work was the economical estimation of GRFs. Therefore, a uniaxial load cell was utilized, along with deep learning, as the three-axis GRF could not be easily estimated with uniaxial data alone. In addition, because the employed sensors are uniaxial, we believe that it will be feasible to use various sensors in the future, such as FSR, piezo-type, and LVDT. Our developed model enables the direct estimation of the three-axis GRF if the sensors are positioned on the 1st metatarsal, the 5th metatarsal, and the heel, the data are collected during walking, and the units are converted to the same format as the data employed in our study. Furthermore, data gathered via insoles constructed with FSR arrays [5,11] can also be utilized to estimate GRF if only the sensor values from the metatarsal and heel areas are employed. However, it should be noted that we employed load cells in this study to mitigate any errors stemming from the flexible nature of the FSRs [16,17]. As a result, we did not factor in any biases, such as voltage drifts, which could result in increased error in the estimation outcomes. Nonetheless, our model is noteworthy as it presents a generalizable method for transforming data collected via a minimal number of uniaxial sensors into a three-axis GRF.

The accuracy of this study has been validated to some extent; however, other recommendations must be explored. In this study, only 81 young and healthy participants were chosen. Because many musculoskeletal and neurological system problems, such as aging, falling, stroke, and dementia, can impact gait patterns [15,39,43,44,45], it may be difficult to appropriately predict abnormal gaits, such as entering gaits and dragging gaits, using the model from this study since the GRF form would differ from that of a typical case. This is a restriction caused by the fact that our subjects exhibited typical walking patterns because they are healthy persons. Our model is only accurate for the GRFs that occur when young adults walk at their preferred walking speed on level ground. Future research should incorporate these patient characteristics into their evaluations. An additional concern arises in relation to the point of application of the GRFs within this context. Utilizing the coordinates of each load cell and their respective measurements, the point of application of the GRFs can be determined by applying the equilibrium equation. This approach bears a resemblance to the technique employed by a force plate in ascertaining the coordinates of its center of pressure. Nevertheless, this method is subject to certain limitations that warrant further consideration. Firstly, it cannot be reliably asserted that the measurements obtained from the three load cells accurately represent the overall foot pressure. Secondly, load cell readings tend to be minimal at critical instances, such as during heel contact and toe-off, which could render the derived equation susceptible to errors. Thirdly, given that the load cell is affixed to the shoe, the calculation may be sensitive to the brief moments during which the foot disengages from the load cell. We acknowledge these limitations within the scope of our current study and intend to address them in subsequent research endeavors. In addition, the number of datasets used in this study was 109, which is less than the number of datasets used in other studies that used deep learning. Given that the number of training datasets and the accuracy of deep learning are often related, we anticipate that this approach can be enhanced by acquiring more data through additional experiments.

## 5. Conclusions

This work developed seq2seq LSTM and shoes with three load cells for estimating three-axis GRF. The computed GRF matched the observed GRF from force plates well, with respective correlation values of 0.97, 0.96, and 0.90, and respective root mean square errors of 65.12N, 15.50 N, and 9.83 N for the vertical, anterior–posterior, and medial–lateral directions, as well as mid-stance timing errors of 5.61% based on the full gait cycle and 9.32% based just on the stance time. The Bland–Altman analysis showed good agreement for the max vertical GRF. The proposed shoe with three uniaxial load cells and seq2seq LSTM can be utilized for estimating the 3D GRF in an outdoor environment with level ground and/or for gait research in which the subject takes several steps at their preferred walking speed, and hence can supply crucial data for a basic inverse dynamic analysis.

## Figures and Tables

**Table 1:** Characteristic of EzForce-1D [31].

Characteristic	Value
Capacity	980.70 N
Linearity	1.00%
Output	5.0 V
Tolerance	14,700.00 N
Voltage/force coefficient	441.00~637.00 N/voltage
Weight	27.10 g (with 1.0 m shield wire)
Dimension	Radius: 30.00 mm, height: 6.90 mm
Material	Aluminum

**Table 2:** The correlation coefficients and RMSEs between estimated and measured GRFs and the mid-stance timing error.

Result	Axis	Value
Correlation coefficient	Vertical	0.97
	AP	0.96
	ML	0.90
RMSE (N)	Vertical	65.12
	AP	15.50
	ML	9.83
Mid-stance timing error (abs)	0.06 s	

**Table 3:** Results of Bland–Altman analysis on test datasets.

Variables	LLoA, ULoA (N)	LoA 95% CI (N)	Mean Difference 95% CI (N)
Max vertical	−146.50, 212.10	−220.70, 286.20	−10.02, 75.61
Max AP	−32.90, 48.28	−49.69, 65.06	−2.00, 17.38
Max ML	−7.16, 22.22	−13.24, 28.30	4.03, 11.04

ULoA: upper limits of agreement; LLoA: lower limits of agreement; CI: confidence interval.

**Table 4:** Difference in accuracy according to the timing of the pressure on the 1st and 5th metatarsal. High correlation coefficients and low RMSEs were observed in the group with different timing, which represents a relatively common pattern.

Result	Axis	Value	
		Group with Similar Timing	Group with Different Timing
n		5	15
Correlation coefficient	Vertical	0.96	0.98
	AP	0.94	0.96
	ML	0.82	0.92
RMSE (N)	Vertical	99.25	53.73
	AP	16.71	15.09
	ML	11.27	9.35

## Data Availability

Data sharing is not applicable.

## Supplementary Materials

The following supporting information can be downloaded at: https://www.mdpi.com/article/10.3390/s23073428/s1, Table S1: Hyperparameters used in three-axis GRF estimated with seq2seq LSTM model; Table S2: Computer spec; Table S3: Time required for training with 1000 epochs; Figure S1: Learning cost.
