# Supplementary material for: A Deep Learning Model for 3D Ground Reaction Force Estimation Using Shoes with Three Uniaxial Load Cells
Source: Sensors (Basel). 2023 Mar 24;23(7):3428. doi: 10.3390/s23073428 (PMC10099259; doi:10.3390/s23073428)

Supplementary Material

A Deep Learning Model for 3D Ground Reaction Force Estimation using Shoes with Three Uniaxial Load Cells

This supplementary material includes contents which are not included in the main paper due to space limit.

A. Hyper-parameters

Table S1 presents the hyper-parameters used in Section 2.4 of the main paper.

Table S1. Hyper-parameters used in 3-axis GRF estimate seq2seq LSTM model.

**Input data size**	**1st Layer size**			**2nd Layer size**		
	Size	State Activation Function	Gat Activation Function	Size	State Activation Function	Gat Activation Function
200	200	Tanh	Sigmoid	50	Tanh	Sigmoid

**3rd Layer size**			**Dropout**	**Output layer**	
Size	State Activation Function	Gat Activation Function		Size	Loss Function
200	Tanh	Sigmoid	0.5	200	Mean Squared Error

**Learning Rate**	**Optimizer**	**Epochs**	**Mini Batch Size**
0.01(constant)	Adam	1000	32

B. Computer spec

Table S2 is the computer specifications used to train the seq2seq LSTM model in section 2.4 of the main paper.

Table S2. Computer spec.

**Operating system**	**System Manufacturer**	**System Model**	**BIOS**
Window 10 Enterprise	Gigabyte Technology Co., Ltd	X570 GAMING X	F3

**CPU**	**Memory**	**GPU**	
AMD Rygen 7 3700X 8-Core Processor	32,716MB RAM	Model	Total Memory
		NVIDA GeForce RTX 2080	24,360 MB

C. Learning cost

Table S3 shows the time required to learn the seq2seq LSTM model in section 2.4 using the computer in Table S2. And Figure S1 is the learning cost

Table S3. Time required for training with 1000 epochs.

**Learning Time**	1 minute 37 seconds

Figure S1. Learning cost.

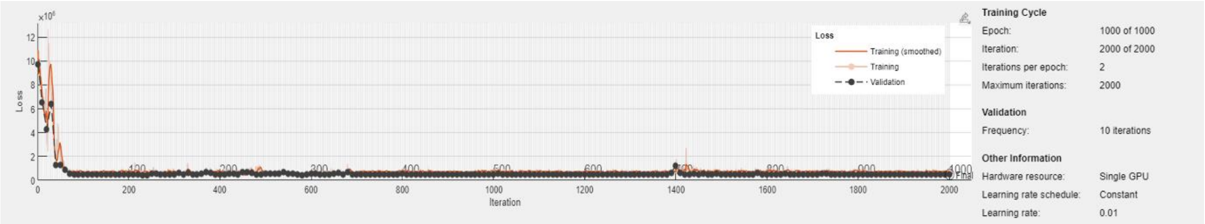

Supplement: Supplementary file 1 [file sensors-23-03428-s001.zip › sensors-2197997-supplementary.pdf]
